# Supplementary material for: Transcriptome Analysis Reveals Vimentin-Induced Disruption of Cell–Cell Associations Augments Breast Cancer Cell Migration
Source: Cells. 2022 Dec 13;11(24):4035. doi: 10.3390/cells11244035 (PMC9776984; doi:10.3390/cells11244035)
Supplement: Supplementary file 1 [file cells-11-04035-s001.zip › DEGs and enrichment analysis.zip!/DEGs and enrichment analysis/WT_VIMvsVector KEGG/ALL/WT_VIMvsVector_ALL.pathway_map.zip!/WT_VIMvsVector_ALL.pathway_map/src/hsa03030.html]

hsa03030


- hsa:2237

- Up regulated genes

ENSG00000168496(1.90827604155967)

- hsa:5982

- Up regulated genes

ENSG00000049541(1.74260731101806)

- hsa:4174

- Up regulated genes

ENSG00000100297(2.27874078513338)

- hsa:4171

- Up regulated genes

ENSG00000073111(2.4296698586728)

- hsa:23649

- Up regulated genes

ENSG00000014138(1.56035482559907)

- hsa:5557

- Up regulated genes

ENSG00000198056(1.89591645158157)

- hsa:5424

- Up regulated genes

ENSG00000062822(2.04128751430151)

- hsa:5425

- Up regulated genes

ENSG00000106628(1.88800442941233)

- hsa:4176

- Up regulated genes

ENSG00000166508(2.12068744963158)

- hsa:2237

- Up regulated genes

ENSG00000168496(1.90827604155967)

Close
